# The Salop Infirmary

**Published:** 1903-12-05

**Authors:** 


### THE SALOP INFIRMARY.

At the recent Anniversary Meeting of the Salop Infirmary, Shrewsbury, at which the Earl of Bradford presided, the following report was presented:—

The report stated that the directors had invited Sir Henry Burdett, K.C.B., to advise them as to the management and cost of the infirmary. Sir Henry Burdett's principal recommendation was to raise the rate of subscriptions giving the privilege of recommending an in-patient from £1 1s. to £2 2s. That was approved. The directors had the utmost satisfaction in announcing that a large number of subscribers of £1 1s. had increased their subscriptions to £2 2s, and others in like proportion; while ever since the attention of the county was directed to the real needs of the infirmary, donations and new subscriptions had been constantly coming in—a sufficient proof that the call only needed to be made to elicit a hearty response. But a word of caution and explanation was necessary lest the altogether changed appearance of the balance-sheet should be misunderstood. It would be noticed that the year, which ended with June 30th, 1903, closed with a balance to credit of £791 14s. in place of the adverse balance of £2,112 0s. 8d. due to the treasurer on June 30th, 1902. That was mainly due to the directors having withdrawn £2,200 of the mortgage invested on property belonging to Shrewsbury School, in order to pay off the deficit which had been accumulating for several years past, and it must be remembered that that entailed a loss of annual income from investments of £71 10s. There was no reason, therefore, for assuming that the infirmary was even yet in a sound financial condition, or that there could be any slackening of the laudable efforts that had been made during the last twelve months to increase its income. That no reduction in expenditure was possible was evident from those paragraphs in Sir Henry Burdett's report, in which he said: "The Infirmary is in a sound position with regard to its housekeeping, whether judged by comparing one year with another, or by what observation has shown to be a fitting expenditure"; and, again, on a comparison of the cost per bed occupied at the Salop Infirmary under various heads with the cost of six provincial general hospitals, "it will be seen on reference to the tables that the average cost per bed occupied, and the average cost of each in-patient at the Salop Infirmary compare very favourably with that at the other institutions included in the tables. We may, therefore, conclude that, so far as the expenditure is concerned, the Salop Infirmary is on the whole administered upon a basis which, if it has a fault, errs on the side of over-economy." It was, therefore, in the endeavour to obtain an increased permanent income, especially by annual subscriptions, donations, and church collections, that all friends of the Salop Infirmary must unite. The directors wished to record their gratitude to Sir Henry Burdett for his assistance, and for placing his great experience in hospital management at their disposal, and pointed out that his fee of fifty guineas, which appeared in the accounts, was given back to the infirmary in the form of a benefaction of fifty guineas, which made him a trustee for life.

The managers of the Salop Infirmary are to be heartily congratulated upon the courage with which they faced the difficult position in which they were placed, and the success which has attended their efforts to establish their institution on a sound footing.

